# Antimicrobial peptides with selective antitumor mechanisms: prospect for anticancer applications

**DOI:** 10.18632/oncotarget.16743

**Published:** 2017-03-31

**Authors:** Berthony Deslouches, Y. Peter Di

**Affiliations:** ^1^ Department of Environmental and Occupational Health, Graduate School of Public Health, University of Pittsburgh, Pittsburgh, PA, USA; ^2^ Department of Microbiology and Molecular Genetics, School of Medicine, University of Pittsburgh, Pittsburgh, PA, USA

**Keywords:** antimicrobial peptides, anticancer peptides, host defense peptides, antitumor peptides, cationic peptides

## Abstract

In the last several decades, there have been significant advances in anticancer therapy. However, the development of resistance to cancer drugs and the lack of specificity related to actively dividing cells leading to toxic side effects have undermined these achievements. As a result, there is considerable interest in alternative drugs with novel antitumor mechanisms. In addition to the recent approach using immunotherapy, an effective but much cheaper therapeutic option of pharmaceutical drugs would still provide the best choice for cancer patients as the first line treatment. Ribosomally synthesized cationic antimicrobial peptides (AMPs) or host defense peptides (HDP) display broad-spectrum activity against bacteria based on electrostatic interactions with negatively charged lipids on the bacterial surface. Because of increased proportions of phosphatidylserine (negatively charged) on the surface of cancer cells compared to normal cells, cationic amphipathic peptides could be an effective source of anticancer agents that are both selective and refractory to current resistance mechanisms. We reviewed herein the prospect for AMP application to cancer treatment, with a focus on modes of action of cationic AMPs.

## INTRODUCTION

Despite unprecedented successes in the field of medicine in the last fifty years, cancer remains a serious threat to human survival [1–4]. Chemotherapeutics, in combination with or in addition to surgery and radiotherapy, play an important role in increasing life expectancy of cancer patients [5–9]. Tumors are clones of rapidly dividing cells unregulated by normal mechanisms of growth suppression. Chemotherapy aims at interfering with this uncontrolled process of cell division [10, 11]. However, many cancer drugs typically lack specificity to transformed cells [12–14]. Consequently, they also kill healthy cells undergoing rapid proliferation resulting in toxic side effects. Another limitation of chemotherapy is the development of resistance by tumor cells [15–17]. Thus, a more effective alternative could be other classes of drugs with the property to specifically target cancer cells without toxicity to normal cells. One more requirement is a lower tendency for development of resistance against such drugs compared to conventional chemotherapeutics. AMPs are an untapped resource with low propensity to elicit development of resistance by its target and to display toxicity to healthy cells undergoing rapid proliferation [18–20]. AMPs are an essential component of the host innate immunity [21–23]. Although they display considerable diversity in both primary and secondary structures, [24–27] the cationic amphipathic motif is a typical structural feature of AMPs and an important determinant of antimicrobial functions [28–31]. While AMP investigations have been largely focused on antimicrobial properties, there is increasing evidence that AMPs display antitumor functions, perhaps in the context of a multifunctional host defense system of multicellular organisms. Alternatively, the antitumor property could be a “side-activity” not necessarily implicated in the natural selection of the common cationic amphipathic structure of AMPs. We reviewed herein the potential of AMPs for application to antitumor therapy as anticancer peptides (ACPs) with novel mechanisms. Broad structural and functional properties of AMPs not pertaining to selective action against cancer cells and antitumor efficacy are reviewed elsewhere [32–39].

### Basis for antitumor property of AMPs: selective recognition of cancer cells via electrostatic interactions

**Figure 1:**
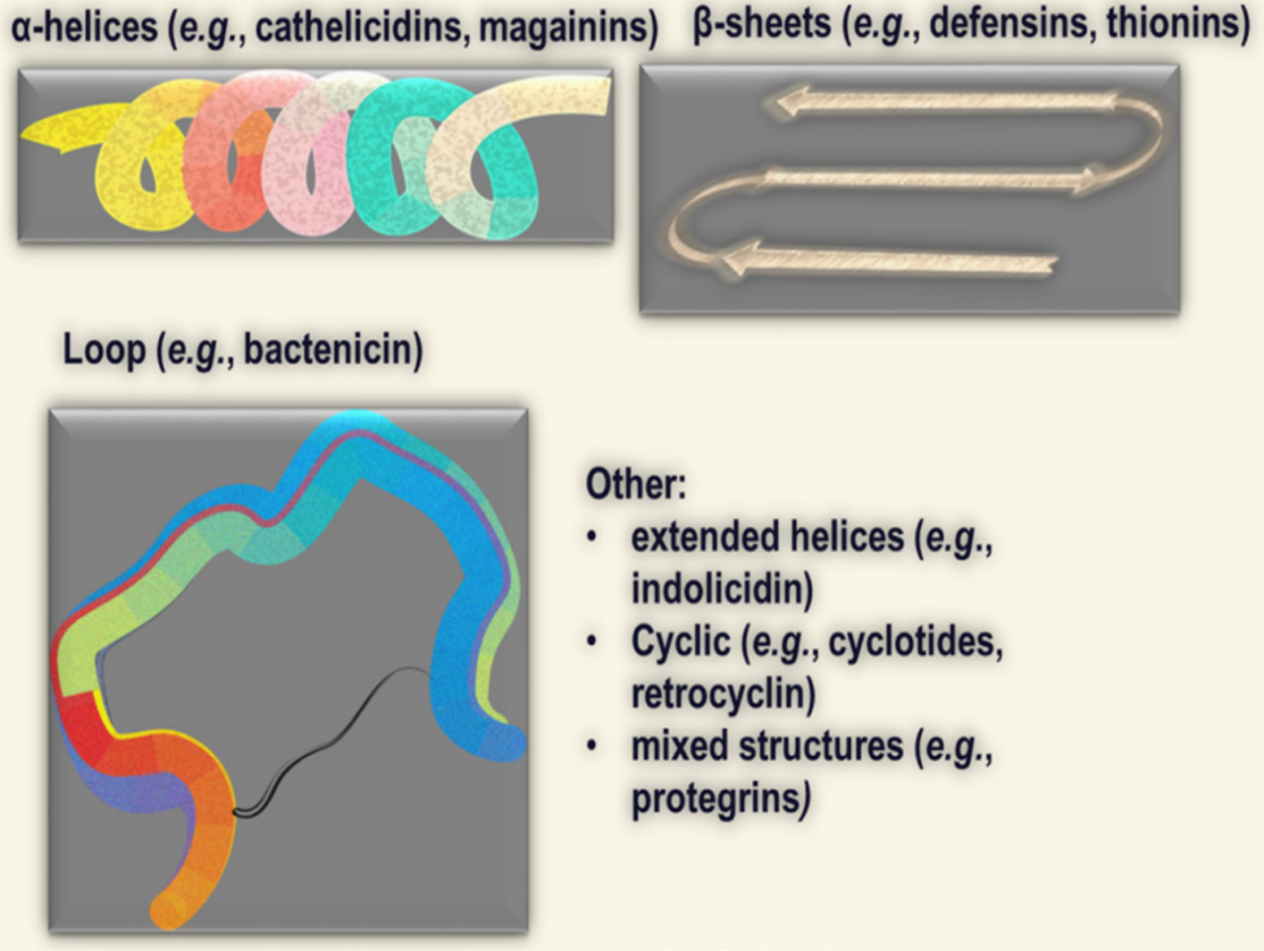
Main structural classes of cationic antimicrobial peptides (AMPs)

Most AMPs are short (typically 10 to 50 residues long) cationic peptides with an amphipathic structure [40–48]. They are structurally diverse in both amino acid compositions and secondary structures (α-helix, β-sheets, extended helix, and loop) (Figure 1). There are multiple reviews of AMP structures, [49–52] which we do not discuss here. AMPs generally recognize their target via electrostatic interactions with negatively charged lipids on cell membranes [53–56]. Because these interactions are not mediated by specific receptors, conversion of L to D enantiomers does not necessarily disrupt the binding capacity of AMPs as shown by Papo and others [57–59]. AMPs display strong interactions with bacterial membranes due to high density of electronegative charges on the bacterial surface, such as lipopolysaccharide (LPS) on the outer membrane of gram-negative bacteria [60–63] or lipoteichoic acid (LTA) on gram-positive bacterial membranes [64–68]. Of note, there is no consensus sequence for binding activities, as cationic AMPs (typical charge of +2 or more) of all types of secondary structures (α-helix, β-sheets, loop alone or in combination; Figure 1) and diverse primary sequences with different positive charges are able to recognize their microbial targets [69–73]. It is, therefore, evident that the cationic amphipathic motif is the main requirement for activity whereas the primary sequence determines specificity or spectrum of activity. While AMPs may display toxicity to mammalian cells, toxic concentrations are commonly one log of magnitude higher compared to minimum inhibitory concentrations against bacteria [74, 75]. Hence, it is logical to predict that AMPs with promising pre-clinical data will display a minimum therapeutic index (maximum tolerated dose/minimum therapeutic dose) required for efficacy in specific applications.

**Figure 2:**
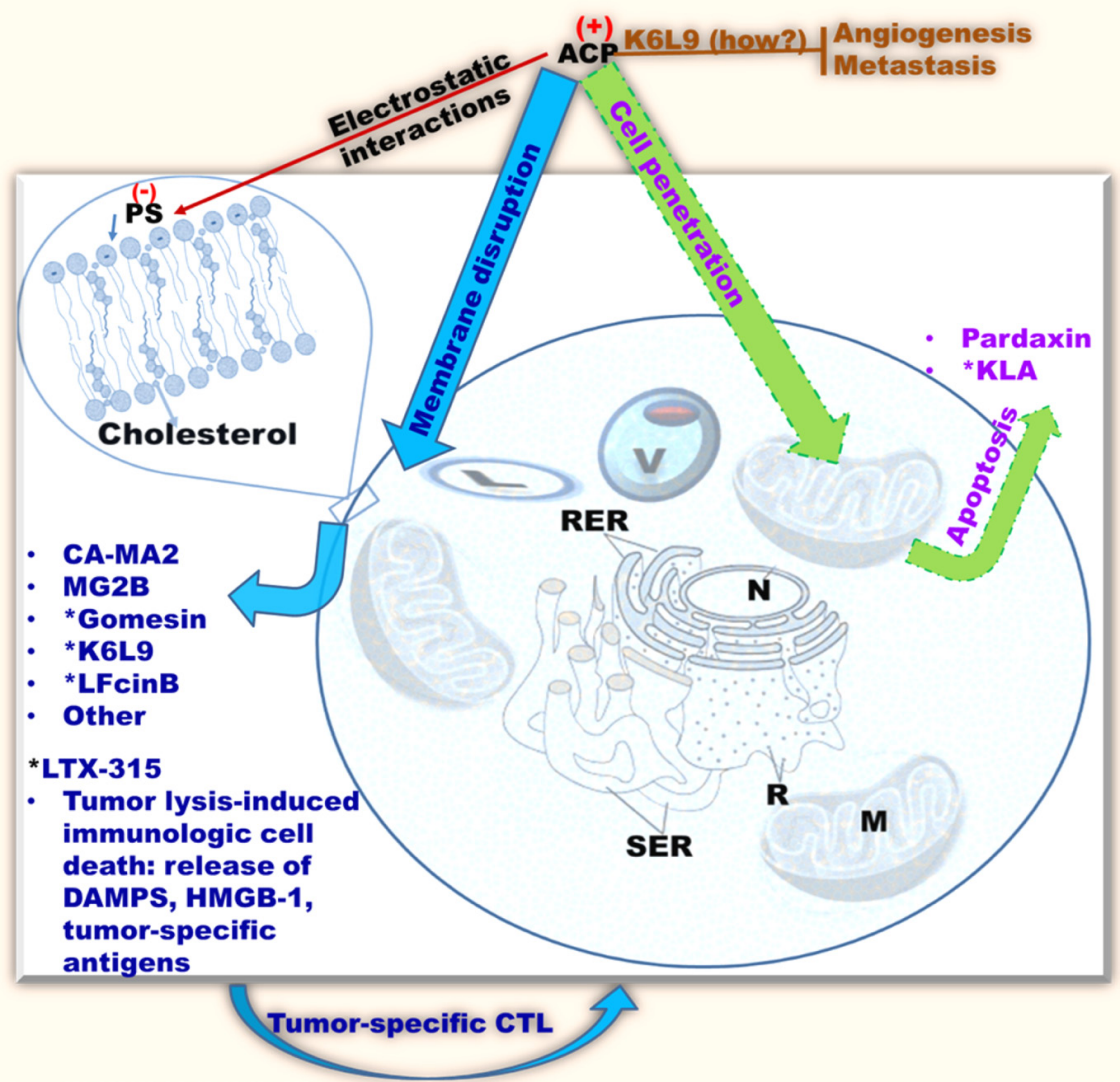
Common antitumor mechanisms of cationic AMPs classified as ACPs ACPs (anticancer peptides), or cationic AMPs with anticancer properties, selectively recognize cancer cells by electrostatic interactions with negatively charged phospholipids on the surface of eukaryotic cells [e.g., PS (phosphatidylserine)]. Some ACPs demonstrate *in vivo* efficacy (e.g., *MGB2, Gomesin, K6L9, LTX-315); ACPs tend to kill cancer cells by membrane perturbation (blue/cyan), although some (e.g., KLA, Pardaxin) may penetrate the target cell and disrupt the mitochondrial membrane resulting in apoptosis (green/purple). (How?), mechanism unclear; brown perpendicular bar, inhibition; CTL, cytotoxic T-Lymphocytes; M, mitochondria, SER, smooth endoplasmic reticulum; RER, rough endoplasmic reticulum; R, ribosomes; N, nucleus; V, vacuole; L, lysosome; only cholesterol and other lipids are shown; membrane proteins are omitted for clarity.

Some AMPs specifically target tumor or cancer cells because transformed cells generally incorporate phosphatidylserine (PS, 3–9% of the total amount of phospholipids) in the outer leaflet of the plasma membrane (Figure 2) [76–78]. PS is usually found on the inner leaflet of the cytoplasmic membrane of normal mammalian cells. However, it can be transferred to the outer leaflet of the plasma membrane of cells undergoing apoptosis, which disrupts the asymmetry observed for normal mammalian cell membranes [79, 80]. Hence, this change in asymmetry is typically shared by both apoptotic cells and several types of cancer cells and facilitates recognition and clearance of these cells by monocytes [81–83]. Other factors that may contribute to elevated negative charges on cancer cells include heparin sulfates, [84–86] and O-glycosylated mucins on the surface of tumor cells [87–90]. However, the density of electronegative charge (due to a single phosphate group on PS) on cancer cells is relatively lower compared to negative charges (due to multiple phosphate groups on LPS, LTA, in addition a phosphate group on PS) on bacterial cell membranes. As a result, AMP affinity for cancer cells is inherently weaker than the affinity for bacteria. Of note, electronegative charges do not always enhance activity. As shown by Fadnes *et al*. (2009), Cell surface heparin sulfate inhibits the bovine AMP lactoferricin by sequestering the peptide away from the lipid membrane [91]. Cancer cell membranes display other properties that may facilitate killing by AMPs compared to normal cell membranes. Some transformed cells may incorporate lower levels of cholesterol in their membranes, enhancing fluidity. For instance, cell membranes of human leukemia and lung cancers display increased fluidity due to a lower level of cholesterol in their membranes compared to membranes of normal leukocytes and pulmonary cells [92–94]. This increase in membrane fluidity may potentiate lytic effects of AMPs as in the case of cecropins and other peptides [95–97]. Conversely, some cancer cells incorporate elevated cholesterol levels as part of lipid rafts (*e.g*., prostate cancer) in their membranes compared to normal cell membranes [98, 99]. Therefore, the role of cholesterol content in cationic ACP activity against cancer cells remains unclear. For instance, some enveloped viruses are susceptible to AMPs (*e.g*., LL37 activity against herpes simplex and influenza viruses) although they incorporate high cholesterol content (lipid rafts) in their membranes [100, 101]. Another interesting property of cancer cells, which may enhance AMP binding, is the increase in surface area with increasing number of microvilli [102, 103]. Upon binding to cancer cells, AMPs may either disrupt the membrane or penetrate the cell and attack the mitochondria leading to apoptosis. The defensins (29–45 amino acids long), an important class of Cys-rich antimicrobial peptides (β-sheets, Figure 1), were among the first AMPs to be discovered and to demonstrate antitumor activity. Although these AMPs have been isolated from different species including plants, [104, 105] the α-(Cys1-Cys6, Cys2-Cys4 and Cys3-Cys5 bridges, with Cys residues numbered based on location from the N-terminus) and β-defensins (Cys1-Cys5, Cys2-Cys4 and Cys3-Cys6 bridges) synthesized in humans are the most studied defensins to date [104–109]. Antitumor activities of α-defensins, notably the human neutrophil peptides (HNP) 1–3, have been demonstrated via both membranolytic and apoptotic mechanisms as well as inhibition of neovascularization required for tumor growth [110, 111]. However, the HNPs also kill normal cells such as fibroblasts, epithelial cells, and leucocyte. Similarly, the plant defensins also display a lack of selectivity towards tumor cells [112–115]. As a result, the defensins are generally not efficient ACP therapeutics, or they require structural optimization to achieve antitumor selectivity. We examined hereafter the antitumor properties of AMPs derived from animals based on two sets of mechanisms, selective plasma membrane disruption or non-membranolytic cytotoxicity.

### Evidence for selective membrane disruption

One of the earliest indications that AMPs may be a source of anti-tumor therapy is a study by Cruciani and colleagues [116]. Discovered by Michael Zasloff, the magainins are a family of AMPs with broad-spectrum antibacterial activity found in the African frog *Xenopus larvae* [117–119]. Cruciani *et al*. showed that magainin 2 and its analogues demonstrated activity selectively against both hematopoietic and solid tumor cells. The cytotoxic effects of the magainins were rapid (within 1 hour) at a concentration as low at 12 μg/mL and were not observed against normal lymphocytes even at up to 200 μg/mL. The peptides induced ion channels leading to leakage of Na^+^, K^+^, and Cl^−^ ions. The cytotoxic effects of the peptides were abrogated when the electrical gradient was eliminated prior to peptide exposure, indicating the membrane potential is essential to the peptide activity. Exposure of the mitochondria to the peptide resulted in inhibition of respiration and leakage of glucose through the peptide-induced channels [120]. Subsequently, an interesting link was established between two independent discoveries [the Cecropins (insect) and magainins (frog)] by Boman and Zasloff, respectively [119, 121, 122]. In this study a hybrid between Cecropin A and magainin 2 (CA-MA-2, KWKLFKKI-P-KFLHSAKKF-NH2) was constructed with three more derivatives based on proline substitutions in the hinge region [123]. These investigators discovered that the activities against several tumor cell lines were enhanced compared to toxicity to erythrocytes and primary fibroblasts. The cationic peptide CA-MA-2 displayed no detectable hemolysis and cytotoxicity against the primary cell NIH-3T3 fibroblast at concentrations up to 100 μM. In contrast, the 50% inhibition concentration (IC50) against several tumor cell lines was as low as 20 μM. it is important to note that antimicrobial activity was always higher than the anti-tumor effects, suggesting that these AMPs could be further optimized specifically for enhanced antitumor properties. Disruption of large phosphatidylcholine (PC)/phosphatidylserine (PS)-based unilamellar (mixed PC-PS) vesicles by CA-MA-2 indicated a membrane perturbation mechanism, although it would have been informative for the investigators to include the parent peptides Cecropin A and Magainin 2 in this study. Another example of cell membrane disruption was more directly demonstrated by the AMP chrysophsin-1 (FFGWLIKGAIHAGKAIHGLI) [124]. Using fluorescent, scanning and transmission electron microscopy, combined with LDH release, the investigators showed convincingly that the cationic amphipathic peptide disrupted the plasma membrane of several cancer cell lines at much lower concentrations compared to the CA-MA-2 peptide. Cancer cell death by apoptosis was ruled out as caspase expression and activities were not affected by chrysophsin-1.

**Figure 3:**
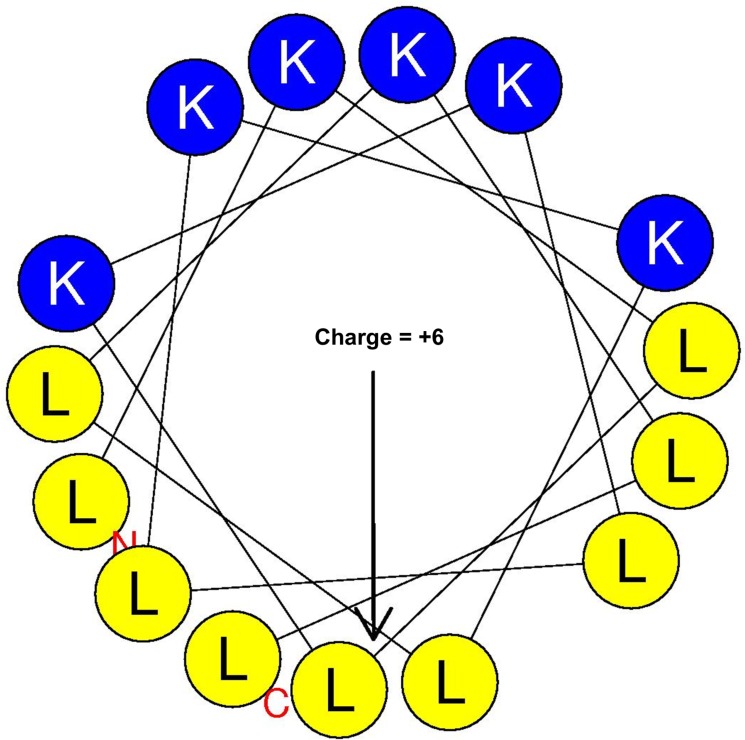
Helical wheel analysis of the engineered AMP K6L9 designed by Papo et al. [57] The peptide was modeled to form an idealized amphipathic helix with only two amino acids; a structural optimization strategy that has been shown to enhance antimicrobial functions, now applied to antitumor properties as well. Arrow indicates direction of the hydrophobic moment. Structural motifs: yellow, hydrophobic; blue, cationic.

While these previous studies show great promise, they fell short of demonstrating anti-tumor efficacy *in vivo*. In addition, AMP activity against cancer cells is much lower than their antimicrobial activity. Hence, two main problems remained to be addressed: (1) the limited anti-tumor activity and (2) specificity toward tumor cells. Both limitations can be overcome if the specificity of AMPs towards tumor cells is enhanced. The strength of AMP interaction with cancer cells may affect both activity and specificity. Hence, in 2011 Liu and colleagues published an elegant study addressing this limitation. They reasoned that linking an AMP to a cancer-homing peptide would enhance specificity and activity against cancer cells. Isolated from frog skin, Bombesin appeared to be a good candidate as the 14-residue peptide recognizes a variety of human cancer cells. The question was which AMP would be the best choice for this experiment? Magainin II (MG2), also derived from frog skin, was one of the most studied AMPs at the time. In fact, the magainins are likely to become the first classical family of AMPs to be used clinically as the derivative pexiganan (cream, 0.08%) is currently in phase III clinical trials in patients with mild infections of diabetic foot ulcers [125–127]. Hence, MG2 was linked to Bombesin (MG2B, GIGKFLHSAKKFGKAFVGEIMNSGG-QRLGNQWAVGHLM). The MG2B peptide displayed higher cytolytic effects compared to MG2. In fact, the *in vivo* efficacy of MG2B was demonstrated in mice bearing MCF-7 tumor grafts. With a daily intratumoral injection of MG2B (20 mg/kg) for 5 days, there was a significant reduction of the tumor size in mice [128]. There are several other examples of membranolytic effects of ACPs, including the use of the ACP gomesin (ZCRRLCYKQRCVTYCRGR) in a cream formulation for successful topical treatment in mice [129, 130]. Importantly, one of the most significant anti-cancer effects of a membranolytic ACP was demonstrated by Papo and colleagues (Figures 2 and 3) [131–133]. The investigators constructed a D-enantiomer of an engineered ACP K6L9. With a daily dose of the peptide (9 mg/kg) injected systemically every other day for a total of nine doses, immunodeficient mice implanted with both breast and prostate metastatic cancers were protected against malignant disease. The tumors became necrotic, and the density of the tumor-induced neovascularization was significantly reduced. The selective binding of the ACP to the negatively charged PS and cytoplasmic membrane depolarization were also demonstrated. The ACP D-K6L9 (LKLLKKLLKKLLKLL) is the most successful demonstration of the systemic anticancer efficacy of an ACP to date [58]. Notably, D-K6L9 is made of only two amino acids. As natural AMPs work in the context of a multifunctional immune system, they are more efficient at protecting the host against disease than they are at curing established illnesses (more preventive than therapeutic). Hence, it is logical to engineer AMPs with more optimized structures for therapeutic applications as opposed to using AMPs as they are produced in nature. We predict that the success of AMP engineering will be facilitated by the establishment of a definitive framework for distinguishing the unique role of the cationic from that of the hydrophobic domain in selectivity toward the target versus host cells. Once a guideline for selective killing of different target cells is established, it will then be possible to design AMPs with enhanced therapeutic index with less trial and error. In the case of the peptide K6L9, the investigators use the principle of amphipathicity to design a 15-residue AMP (charge = +6) with an idealized amphipathic helix (Figure 3). In addition, the use of D-amino acids enhances stability, addressing a concern for lability of AMPs [58, 132–134]. This is an important example of how AMP engineering can lead to enhanced results. Yet, the most advanced (in terms of clinical trials) cationic ACP is not D-K6L9. Over the last two decades, there has been an increasing interest in bovine lactoferricin (LFcinB). LFcinB is derived from the natural milk protein bovine lactoferrin (LFB) by pepsin digestion [135–139]. In addition to its antimicrobial properties, [140–143] LFcinB demonstrates enhanced activities against cancer cells compared to normal mammalian cells [139, 144, 145]. Importantly, high-resolution imaging revealed a membranolytic mechanism [146]. These studies reached a turning point when Eliassen and colleagues (2006) began to examine the *in vivo* efficacy of LFcinB in mice, demonstrating the growth inhibition of neuroblastoma xenografts [147]. While the peptide could be localized in the mitochondria with caspase activation, the lytic effects on the cytoplasmic membrane represented the primary cytotoxic mechanism, as pan-inhibition of the caspase enzymatic cascade could not reverse the cytotoxic effects of the peptide. These studies led to further structural optimization using 3,3-diphenylalanine (Dip) substitutions for Trp at specific positions of the 9-mer template. These investigations resulted in enhanced selectivity (IC50 below 5–10 μM) against a variety of tumors compared to normal cells. More importantly, these studies revealed a cytolytic-immunogenic dual cell death mechanism [148–156]. Lysis of the tumor induced the release of danger-associated molecular patterns (DAMPs) including high mobility group box protein 1 (HMGB1, reviewed by Frank *et al*., 2015 [157]) in addition to antigens from the dead tumor cells. As potent stimulators of immune responses, HMGB1 potentiated A20 lymphoma-specific activation of immature dendritic cells with subsequent generation of tumor-specific cytotoxic T-lymphocytes and tumor cell lysis. The immunogenic cell death was further confirmed by the adoptive transfer of syngeneic A20 lymphoma-specific CTLs and resulting protection of immunodeficient mice from tumor implants. Further, LTX-315-treated mice in remission from the tumor were protected from a second challenge by the syngeneic tumor and not by a different tumor type, indicating the specificity of the immunogenic cell death. Hence, the potent derivative LTX-315 was selected for advanced preclinical studies. LTX-315 is now (2016) in phase 1 clinical trial as described later. The study of LTX-315 is yet another illustration of how natural AMPs can be optimized for specific clinical applications.

### Non-membranolytic mechanisms of ACP

The ACP K6L9, while exerting a direct membranolytic effect on cancer cells, demonstrates anti-angiogenic and anti-metastatic effects, an anti-tumor mechanism that remains to be elucidated. Another common anti-cancer mechanism is the potentiation of apoptosis in cancer cells by ACP [158–160]. While ACPs selectively interact with cancer compared to normal cell membranes, some AMPs may both perturb the membrane and penetrate the cells while other non-lytic ACPs may simply traverse the membrane and access the intracellular compartment. In both cases, the ACP disrupts the mitochondria (as expected because of the bacterial origin of mitochondria) and induces programmed cell death. This apoptotic mechanism is demonstrated by swelling of the mitochondria, disruption of the mitochondrial membrane, translocation of PS to the surface of the cell, and stimulation of apoptotic markers (*e.g*., caspase enzymatic pathway). One of the earliest examples of an apoptotic ACP was reported by Mai *et al*., 2001 [161]. The authors used a KLA repeat AMP [(KLAKLAK)2] conjugated with a transduction peptide (RRQRRTSKLMKRGGKLAKLAKKLAKLAK) by a glycine pacer. The intratumoral injection of the chimeric ACP resulted in the translocation of the peptide into the cytoplasm of the tumor cells, disruption of the mitochondria, and stimulation of apoptotic enzymatic cascade by caspase 3 activation. Other ACPs have the inherent property to penetrate the cancer cells without the need for conjugation with a cell penetrating peptide. An example of such a peptide (GFFALIPKIISSPLFKTLLSAVGSALSSSGGQE) is pardaxin, which induces apoptosis of squamous cell carcinoma cells by caspase 3 activation [162]. In addition, some AMPs may be used as cell penetrating peptides to transfer an anticancer drug into cancer cells. This is the case of the AMP PR39 (RRRPRPPYLPRPRPPPFFPPRLPP RIPPGFPPRFPPRFP) used as a cell penetrating peptide to transfer Stat3 siRNA (the cargo) into breast cancer cells [163–165]. As previously mentioned, another interesting antitumor mechanism is the immunogenic cell death conferring long-lasting protection against future challenges from the same tumor type. Such a mechanism is displayed by the ACP LTX-315, [151] which was selected for advanced preclinical studies. LTX-315 is now in phase 1 clinical trial [151, 155, 156]. Noteworthy is an antitumor mechanism occurring via the lysosomal-mitochondrial death pathway by the defensin Brevenin-2R (*in vitro* activity) [166]. As the anticancer mechanisms of AMPs become increasingly clear, structural optimization for enhanced anticancer potency will be more achievable and, therefore, clinical development more likely to succeed.

Of note, AMPs synthesized in plants are another important source of peptide therapeutics that could be used as anticancer agents. However, for many of these AMPs, the anticancer mechanisms are unclear. AMPs derived from plants are beyond the scope of this review and are reviewed elsewhere [32]. A list of representative AMPs with selective antitumor mechanism is shown Table 1.

**Table 1:** Representative antimicrobial peptides with selective antitumor mechanisms

AMP name	Amino acid sequence	Source	Tumor target	Mechanism	Reference
Alpha-defensin-1	ACYCRIPACIAGERRYGTCIYQGRLWAFCC	Human	HTC/STC	Apoptosis	Xu et al., 2008 [109]
				Antiangiogenic	
BMAP-28	GGLRSLGRKILRAWKKYG	Bovine	HTC	MP/Ca influx	Risso et al., 2002 [158]
				Apoptosis	
Brevenin-2R	KFALGKVNAKLQSLNAKSLKQSGCC	Frog	STC	LDP	Ghavami et al., 2008 [165]
Buforin IIb	RAGLQFPVG[RLLR]3	Frog	HTC/STC	Apoptosis	Lee et al., 2008159
CA-MA-2	KWKLFKKI-P-KFLHSAKKF	Hybrid	STC	MP	Shin et al., 2000 [122]
Cecropin A	KWKLFKKIEKVGQNIRDGIIKAGPAVAVVGQATQIAK	Silk moth	HTC	MP	Hui et al., 2002 [96]
Cecropin B	KWKVFKKIEKMGRNIRNGIVKAGPAIAVLGEAKAL	Silk moth	HTC/STC	MP/Apoptosis	Li et al., 2016 [94]
chrysophsin-1	FFGWLIKGAIHAGKAIHGLI	Red sea bream	HTC/STC	MP	Hsu et al., 2011 [123]
D-K6L9	LKLLKKLLKKLLKLL	Engineered	STC	MP	Papo et al., 2006 [57]
Gomesin	*ZCRRLCYKQRCVTYCRGR	Spider	STC	MP	Domingues et al., 2010 [128]
KLA	RRQRRTSKLMKRGGKLAKL-AKKLAKLAK-(KLAKLAK)2	Engineered	STC	MP	Mai et al., 2001 [160]
lactoferricin B	FKC1RRWQWRMKKLGAPSITC1VRRAF	Bovine	HTC/STC	MP/Apoptosis	Eliassen et al, 2002 [145]
LL37	LLGDFFRKSKEKIGKEFKRIVQRIKDFLRNLVPRTES	Human	Ovarian CA	MP	Chuang et al. 2009 [210]
LTX-315	K-K-W-W-K-K-W-Dip-K	Engineered	HTC/STC	MP/ICD	Haug et al., 2016148
			Phase I/II trial		
Magainin 2	GIGKFLHSAKKFGKAFVGEIMNS	Frog	HTC/STC	MP	Cruciani et al., 1991115]
Melittin	GIGAVLKVLTTGLPALISWIKRKRQQ	Insect	STC	MP	Wang et al., 2009 [56]
MG2B	GIGKFLHSAKKFGKAFVGEIMNSGG-QRLGNQWAVGHLM	Hyprid AMP	MCF-7 tumor	MP	Liu et al., 2011 [128]
Pardaxin	GFFALIPKIISSPLFKTLLSAVGSALSSSGGQE	Fish	STC	MP	Han et al., 2016 [162]

* pyroglutamic acid; HTC, hematological tumor cells; STC, solid tumor cells; MP, membrane permeabilization; LDP, lysosomal death pathway; ICD, immunological cell death.

### Prospect for clinical use of AMPs as anticancer agents

The clinical development of AMPs as ACPs faces some of the same challenges to AMP clinical development as antimicrobial agents. AMPs are not traditional drugs. Because they have multifunctional properties, the adaptation from their natural environment to clinical applications without structural optimization is rather challenging. While AMPs work well in the context of a competent immune system, it is likely that in nature a particular AMP structure is not completely optimized for a single function (*e.g*., antibacterial, antiviral, or anticancer). The field also faces some unfounded criticisms that have hindered support for AMP development. Some of these criticisms are that (1) “AMPs are labile and likely to have poor pharmacokinetic properties”, an assumption based on the peptidic nature of AMPs; (2) “AMPs are expensive to make”; (3) “AMPs are not good drugs” because they do not recognize specific receptors.

**Figure 4:**
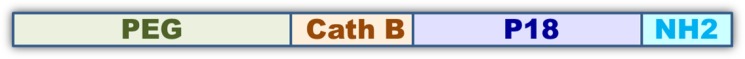
Strategy to improve the PK properties of AMPs adapted from Kelly et al., 2016 [183] The AMP P18 is amidated at the C-terminus. In addition, it is covalently bound to the protease cathepsin B-sensitive linker for the release of the cancer-active drug; this linker is also covalently attached to a polyethylene glycol (PEG) polymer, which is a hydrophilic moiety that serves as a protective shield from protease degradation and drug clearance.

Although stability is an important concern, the first criticism is based on the assumption that all peptides have similar stability and clearance mechanisms. One of the shortcomings of AMPs is the lack of correlation between *in vitro* susceptibility testing and efficacy in animal models, with some exceptions including some of the AMPs discussed in this review and elsewhere [58, 156, 167–169]. Assays establishing correlation between *in vitro* stability and bioavailability in animal models might address this concern. Currently, AMPs with optimized structures are amenable to parenteral administration including systemic, respiratory, intramuscular, intraperitoneal, or subcutaneous unless otherwise contraindicated [167, 168, 170–172]. Although the peptidic nature of AMPs precludes oral delivery, the development of specific delivery systems protecting AMPs from degradation by digestive enzymes may increase intestinal absorption and the feasibility of oral administration. Several strategies can be used to enhance PK properties of AMPs. One approach is the utilization of D-enantiomers to increase stability, [133] although such strategy is only indicated if decreasing peptide clearance enhances therapeutic efficacy and does not potentiate toxic side effects. Other strategies include end-to-end cyclization, C-terminus amidation, pegylation [attachment of polyethylene glycol (PEG) to a molecule], and liposomal delivery. Cyclisation and amidation can confer peptide stability by decreasing susceptibility to protease digestion, as demonstrated by the modification of gomesin and other AMPs [173, 174]. As a principle, all AMPs used in our laboratory are amidated [18, 20, 175, 176]. As shown by the Gumbleton group and others, pegylation of AMPs can result in lower host toxicity without affecting antimicrobial activity [177]. Pegylation enhances the pharmacological properties of a given drug in a number of ways [178–183]. Because it increases hydrophilicity, PEG serves as a shield that protects against protease digestion, prolongs circulation time, and reduces the glomerular filtration rate. An interesting example of AMP pegylation is a recent design by Kelly *et al*., 2016 as shown in Figure 4 [183]. A pro-peptide was designed using the AMP P18 covalently attached to a linker region that is sensitive to the cysteine protease cathepsin B, followed by a PEG region to confer thermodynamic stability to the peptide. *In vitro* studies show that selectivity index can be enhanced against an ovarian carcinoma cell line (A2780). While this strategy is still conceptual, as it requires extensive *in vivo* studies for proof of concept, it shows how a pro-peptide can be designed in combination with pegylation to enhance the PK properties of an ACP or AMP. Another strategy could be the liposomal formulation of AMPs [184–187]. However, one shortcoming is that AMPs are membrane active and could, therefore, bind and disrupt the liposome. Packaging the molecule as an inactive pro-peptide in the liposome could theoretically overcome this problem. One last potential caveat is whether we are able to produce liposomes that can discriminate between the target and normal cells. An AMP delivered inside a eukaryotic cell will probably interact with mitochondria as it would with a bacterial cell, based on similarities between a bacterial cell and mitochondrial membranes. One strategy that is already developed is to tag the lysosome with a cancer cell-specific ligand for targeted delivery of the peptide [188]. Finally, designing AMPs as part of nanoparticles for delivery is another strategy that may improve PK properties of AMPs and should be explored [189–191]. All of these strategies for enhancing PK properties should be considered only when indicated, as AMPs are highly diverse in structure, which affects PK properties.

The second criticism is based on an outdated notion of the highly impractical cost of peptide production. While cost remains a concern, peptide- and protein-based drugs have been used clinically for decades from anti-hypertensive (*e.g*. Lisinopril) [192–196] and anti-diabetic (*e.g*., insulin) [197, 198] to immune, antiviral (*e.g*., fuzeon), [199, 200] antibacterial, [201–203] and hormonal therapy [196]. Modern technology and larger scale synthesis have also significantly reduced the production cost of AMPs. The third concern is based on the fact that AMPs do not select their targets via specific receptors [204]. This nonreceptor-mediated recognition is one of the major reasons AMPs are less likely to invoke selection of resistance compared to current antibiotics/anticancer drugs and the basis for broad selectivity against diverse types of multidrug-resistant microbial pathogens and transformed cells. Lipid-mediated selectivity of AMPs should be considered as a major strength, not a weakness. These misconceptions against AMPs have considerably hampered the progress of their development, particularly AMP engineering for structural optimization in the United States. The success of the ACPs K6L9, LTX-315, and other engineered antibacterial AMPs [20, 167, 168, 175, 176] illustrates the need for AMP engineering and, thus, the establishment of a rational framework for predicting structural determinants of the selective killing of different target cells. Such a guideline for structural optimization, combined with PK-enhancing strategies, would enhance our ability to increase the therapeutic index of AMPs with significantly less trial and error.

Despite the aforementioned challenges, there are a few AMPs being evaluated as anticancer (in addition to antimicrobial) agents in advanced phases of clinical development. The human cathelicidin LL37 is currently (2016) in Phase 2 clinical trial for melanoma (lesions at least 10 mm and not completely resectable) by intratumoral injections in patients with no known immune deficiency, a collaborative effort of M.D. Anderson Cancer Center and National Cancer Institute [205]. The first patients were enrolled in July 2015. LL37 is the single most studied human AMP [206–208]. It is normally found in human skin, reproductive, and respiratory systems and known to have multiple functional properties including (but not limited to) antibacterial, antiviral, antifungal, immunomodulatory, and anticancer activities [209–211]. Another AMP, LTX-315, is in phase 1 trial for PK and efficacy treatment of multiple types of transdermally accessible tumors. This is a lactoferrin-derived lytic peptide that binds and lyses tumor cells. The resulting tumor necrosis leads to enhanced presentation of tumor antigens and induced innate and adaptive immunity against the tumor as described above. This clinical trial started on October 28, 2013, and the last update (December 2016) indicates that LTX-315 is still in phase 1 “Open-label, multi-arm, multi-centre, multi-dose, dose Escalation Study” for exploration of efficacy as monotherapy or in combination with either ipilimumab or pembrolizumab in patients with transdermally accessible tumors” [212]. Noteworthy are other AMPs in clinical trial as anti-infective agents (*e.g*., OP-145 for otitis media and pexiganan for diabetic foot ulcer), but not as ACPs. Toward the goal for clinical applications, it is possible to improve the therapeutic index of current chemotherapeutic agents by considering combination therapy. Combining AMPs with a chemotherapeutic agent may help decrease dosage, which would result in lower toxicity. While the current clinical trial of LTX-315 including combination with immunotherapeutics (Ipilimumab or Pembrolizumab) is a step in the right direction, combination therapy using AMPs and chemotherapeutic agents needs to be explored for possible synergy [183].

### Concluding remarks

Since the discovery of AMPs more than three decades ago, no other class of compounds has matched their versatility as multifunctional compounds. AMPs have the potential to become the only class of drugs that can be used against polymicrobial co-infections (*e.g*., bacterial and viral [213]) and cancer. However, the multifunctionality determined by a typical AMP structure suggests that no single property is completely optimized in natural AMPs in the context of maintaining multiple functions. Rational peptide engineering is essential to AMP development for clinical applications. An important task is to dissect the structural determinants of each property to uncouple each of AMP functions for “application-specific optimization”. Only when such studies are conducted in a systematic way will we begin to significantly explore the clinical potential of AMPs as a diverse class of therapeutics. AMP research has been largely occurring outside of the United States. Despite a vast literature in AMP research, this is still an area that is critically underfunded by the National Institute of Health (NIH). Because of the initial failure of AMPs to reach the Clinique, the resulting bias has largely hindered the advancement of AMP research in the United States. Hence, there is a pressing need for the NIH and pharmaceutical companies to support more AMP research to collect the evidence necessary to assess whether the promise of AMPs will ever come to fruition. As an essential component of the immune system, AMPs warrant such exploration.
